# Cavernosal Abscess Mimicking Ischemic Priapism in a Diabetic Patient: A Report of a Rare Case

**DOI:** 10.7759/cureus.90040

**Published:** 2025-08-13

**Authors:** Bhavyadeep Korrapati, Vijayanand Mani, Velmurugan Palaniyandi, Hariharasudhan Sekar, Sriram Krishnamoorthy

**Affiliations:** 1 Urology, Sri Ramachandra Institute of Higher Education and Research, Chennai, IND

**Keywords:** abscess, diabetes mellitus, impotence, priapism, cavernosa

## Abstract

Cavernosal abscesses are extremely rare and an even rarer precipitant of ischemic priapism. These abscesses may arise due to trauma, intracavernosal injection therapy, foreign bodies, or complications of perianal and perineal infections. Hematogenous spread from distant sources, including dental infections, has also been documented. Diabetic individuals are particularly susceptible due to their immunocompromised status, microvascular disease, and poor glycemic control. Delayed diagnosis risks fibrosis and lifelong erectile dysfunction. A 56-year-old man with poorly controlled type-2 diabetes (HbA1c 10.2 %) presented with fever and painful low-flow priapism of two hours duration. Inspection revealed a perineal abscess that opened and expressed approximately 60 mL of purulent material, resulting in immediate detumescence. A pelvic 3-T MRI performed 40 minutes later demonstrated a T2 hyperintense collection showing diffusion restriction, measuring 7.3 × 5.1 × 7.6 cm, with a volume of 132 cc noted in the left corpora cavernosa and perineum. Intravenous cefoperazone-sulbactam plus metronidazole was started and de-escalated to amoxicillin-clavulanate after cultures grew methicillin-sensitive Staphylococcus aureus. On hospital day 2, a 1-cm perineal incision allowed blunt evacuation and gentamicin-saline irrigation; the drain was removed after 48 hours. Adjuvant tadalafil 5 mg daily and intensified insulin therapy were continued for six weeks. At six months, the patient reported satisfactory erectile function, including normal rigidity and the ability to complete intercourse without the use of pharmacological support. In diabetic men presenting with painful priapism, the presence of a small perineal abscess should raise suspicion of an underlying cavernosal abscess. Early MRI delineation, combined with minimal drainage and culture-directed antibiotics, can eradicate the infection while preserving erectile function.

## Introduction

Corpus cavernosal abscesses usually present with pain, swelling, erythema, and increased local rise of temperature. These are rare but potentially serious infections of the penis [1]. The aetiology of these abscesses includes trauma, foreign bodies, intracavernous injection therapy, or complications of perianal and perineal abscesses. Infection that starts from the cavernosal bodies of the penis leads to local inflammation and tissue damage and eventually results in a cavernosal abscess [1,2]. A cavernous abscess may be secondary to intra-abdominal abscesses, or perineal abscesses may cause priapism. A few case reports have reported that it can spread from distant infections, such as periodontal abscesses [2]. Diabetic populations are at high risk due to the immunocompromised state of microvascular changes and high tissue glycaemic status [3]. Cavernosal abscesses constitute one of the rarest penile infections, with roughly sixty English-language cases since 1725. A 2023 systematic review of MEDLINE, Scopus, and Embase identified 52 cases, with 46% of these cases involving individuals with diabetes. The majority followed trauma, intracavernosal injections or shunt surgery. Low-flow priapism as the index presentation is even less common, documented in only 11 patients [4].

Priapism is a condition defined by a prolonged and painful erection that lasts for hours in the absence of sexual stimulation. It can result from various underlying factors, including trauma, medications, systemic diseases, or, in rare instances, the formation of a cavernous abscess [5]. This infection can disrupt normal blood flow and impair venous drainage, preventing the penis from returning to a flaccid state and potentially leading to priapism [6].

Priapism secondary to a cavernous abscess is due to local ischemia, inflammation, and thrombosis, which affects venous drainage. The mechanism mentioned above will prevent the penis from returning to a flaccid state [1,5,6]. Common bacterial pathogens associated with cavernous abscesses include Staphylococcus aureus, Escherichia coli, and various Streptococcus species [7]. Priapism may be secondary due to direct trauma or haematogenous spread from distant infections from the prostate or perineum, which will add further insult to the ongoing infection [8]. Priapism may be the presenting complaint without prior trauma or apparent signs of an acute illness [1]. Priapism secondary to cavernosal abscess requires a careful clinical examination and appropriate history-taking with required imaging like ultrasound, MRI, and CT scan [7]. Blood cultures and urine cultures are necessary to identify the organism and treat it according to its sensitivity [9]. If the abscess is not evaluated initially and not treated aggressively, this can lead to penile fibrosis, leading to permanent erectile dysfunction [10]. Typically, treatment involves empirical antibiotics, drainage, or surgical debridement to reduce extra or intra-corporeal pressure and restore normal circulation [9,10]. If these patients are treated aggressively initially, they typically recover without complications. If not addressed initially, this can lead to irreversible damage, including permanent erectile dysfunction. Penile fibrosis can lead to tissue necrosis and sloughing, resulting in permanent tissue loss [11]. We present a unique case in which spontaneous perineal abscess rupture led to immediate relief of priapism. This case highlights the critical importance of early recognition and prompt intervention in managing priapism secondary to a cavernous abscess to reduce the risk of long-term complications.

## Case presentation

Patient history

A middle-aged man who has had diabetes for 10 years came to the emergency department with complaints of fever with chills and priapism lasting less than two hours. The patient also complained of pain in the scrotum and perineum. The patient has no history of trauma or injection into the corpora cavernosa or any prior erectile dysfunction.

Clinical examination

**Figure 1:**
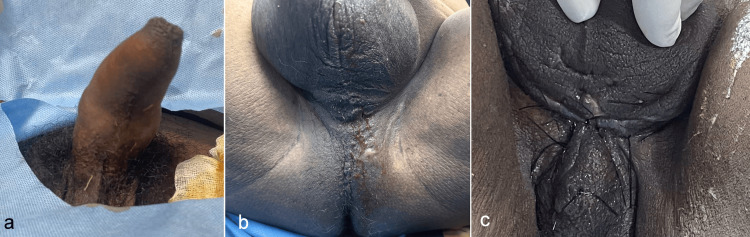
(A) Priapism, (B) An inflamed perineum with pus pointing out, where around 60 cc of pus drained, leading to detumescence, and (C) post-operative image showing a well-healed wound with secondary suturing.

On examination, a fully erect and tender penis was found as shown in Figure 1A. Erythema was present around the perineum at 1 o'clock, with tenderness over the scrotum and the perineal area. A small area with brownish discharge was noted as shown in Figure 1B, and upon palpation of the perineum, a gush of pus, approximately 50 to 60 cc, was observed, which led to detumescence and relief from pain. Baseline laboratory studies showed leukocytosis (18.2 × 10^9/L), thrombocytopenia (92 × 10^9/L), elevated C-reactive protein (128 mg/L), and hyperglycaemia (capillary glucose 370 mg/dL). A cavernous blood-gas sample was deferred to avoid introducing further infection into a decompressed corpus. 

**Figure 2:**
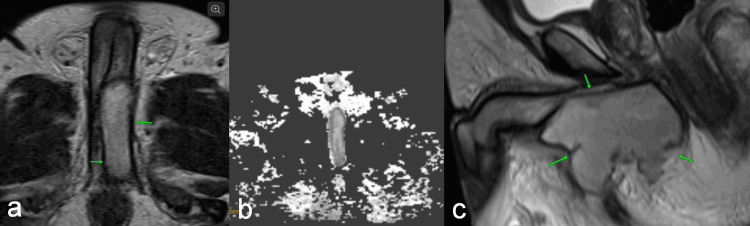
T2 hyperintense image of penis (A), DWI of penis (B) and axial imaging of perineum showing an abscess (C). (A) T2 hyperintense image showing a collection measuring 7.3 x 5.1 × 7.6 cm volume of 132 cc is noted in the left corpora cavernosa, (b) DWI of penis showing abscess extending into corpora cavernosa with no breach into tunica and urethra, and (c) axial imaging of perineum showing abscess collection: Anteriorly extending to the root and penis, extending into the left corpora cavernosa muscle and splaying the urethra and corpora spongiosa. Posteriorly, limited to the perineum with no gross extension into the anal canal. Inferiorly extending into the root of the scrotum and the anterior aspect of the left gluteal cleft. Superiorly seen displacing the left pubococcygeus and puboanalis muscles; however, no extensions beyond it. No extensions into the prostate without tunical or urethral breach. These multiparametric MRI  features represent a left cavernosal abscess with an extension into the perineum. DWI: Diffusion-weighted imaging

Ten minutes later, a bedside Doppler ultrasound was performed, and it demonstrated restoration of antegrade diastolic flow. High-resolution 3-Tesla MRI of the pelvis delineated a T2 hyperintense collection showing diffusion restriction, measuring 7.3 × × 5.1 × 7.6 cm. A volume of 132 cc was noted in the left corpora cavernosa and perineum as shown in Figures 2A-2C. The patient was then started on IV antibiotics and IV fluids.

Treatment

Empirical intravenous cefperazone (1.5 g twice daily) plus metronidazole (500 mg every eight hours) was initiated to cover the broad spectrum of Gram-positive, Gram-negative and anaerobic organisms commonly implicated in cavernosal suppuration. The culture of the purulent aspirate grew methicillin-susceptible Staphylococcus aureus, prompting de-escalation to intravenous amoxicillin for 10 days. On hospital day 2, a small, perennial incision and drainage were performed under spinal anaesthesia. Irrigation with 120 mL of warmed saline containing gentamicin was done, and the defect was closed with interrupted 2-0 nylon with a corrugated tube inset. The procedure was uneventful, and the drain was removed on postoperative day 2, as shown in Figure 1C. The patient was discharged on postoperative day 6 with a seven-day course of oral amoxicillin, daily low-dose tadalafil to mitigate fibrosis, and intensified glycemic control with input from endocrinology.

Outcome and follow-up

By two weeks, the surgical site had healed primarily, and follow-up ultrasound confirmed collapse of the abscess cavity without residual fluid. At six weeks, the patient described consistent return of spontaneous nocturnal erections with no complaints of erectile dysfunction. Duplex Doppler examination demonstrated peak systolic velocities of 38 cm/s bilaterally and preserved diastolic reversal, indicating intact arterial inflow and veno-occlusive competence.

Patient's perspective

At first, the pain and prolonged erection frightened me. I'd never experienced anything like it. After the abscess drained, I felt instant relief both physically and emotionally. The doctors explained the rare infection carefully and worked quickly to treat me, which reassured me and my family. The medications and close follow-up gave me confidence that my health and personal life would recover. Six months later, I'm grateful I can live normally and have intimacy without added support. I appreciate the team's dedication and clear communication throughout.

## Discussion

Cavernosal abscesses remain one of the rarest penile infections, with fewer than 70 well-documented cases in the literature and only eleven describing concomitant priapism. A 2023 scoping review conducted by Hayashi et al. confirmed diabetes mellitus as the leading predisposing factor, accounting for nearly half of all occurrences [4]. Hyperglycaemia disrupts neutrophil chemotaxis and microvascular perfusion, facilitating haematogenous seeding of the relatively avascular corpus cavernosum [12]. Additional triggers, such as intracavernosal injections, penile fracture repair, and shunt procedures, were absent in our patient, highlighting the need for vigilance even in the absence of an antecedent insult. The rapid presentation suggests either an aggressive infectious process or heightened pain sensitivity in the context of poorly controlled diabetes, which is known to impair innate immunity through neutrophil dysfunction and microangiopathy [13].

Low-flow priapism secondary to cavernositis has a distinct pathophysiological profile compared to classic ischemic priapism. Experimental work shows that intraluminal purulence elevates intracavernosal pressure sufficiently to occlude subtunical venous plexuses. At the same time, arterial inflow persists, resulting in painful rigidity described by Burnett et al. [14]. The immediate detumescence observed after bedside drainage in our case directly supports the pressure-mediated veno-occlusive hypothesis, which is similar to the case report explained by Hidaka et al. [1]. This hypothesis suggests that persistently elevated pressure accelerates hypoxia-driven fibro-obliterative remodelling, underscoring the urgency of decompression within the first 24-48 hours [1,14].

Ericson et al. described duplex ultrasound as invaluable for distinguishing between low-flow and high-flow priapism; however, it cannot reliably differentiate between an abscess and a hematoma. MRI, with its superior soft-tissue contrast, accurately delineates cavity size, multilocularity, and tunical integrity, thereby guiding a tissue-sparing surgical corridor. Contrast-enhanced CT may serve as an alternative when MRI is unavailable; however, its sensitivity for sub-centimetre cavities is inferior [6]. In our patient, an early MRI (performed 40 minutes post-decompression) enabled a 2 cm perineal window rather than a debilitating corporotomy.

Management paradigms have shifted away from wide corporotomy, which historically carried erectile dysfunction rates approaching 50 %, toward image-guided aspiration or limited incision combined with broad-spectrum β-lactam/β-lactamase inhibitors. A pooled analysis by Hayashi et al. [4], where 14 modern cases (2000-2024) reveal erectile function preservation in 82% and recurrence ≤ 15%. Based on cavity diameter (<3 cm), absence of technical breach and early presentation (<72 h), we adopted a minimalist strategy similar to the conservative, tissue-preserving approach. We emphasised the importance of early intervention and appropriate surgical techniques, similarly described by Hayashi et al. and Sagar et al. [4,15]. Our antimicrobial regimen of cefoperazone-sulbactam plus metronidazole adhered to the European Association of Urology guidelines for the treatment of soft-tissue infections. It was successfully de-escalated once cultures identified methicillin-sensitive Staphylococcus aureus.

Adjunctive measures play a crucial role in functional recovery. Translational studies demonstrate that low-dose phosphodiesterase-5 inhibitors downregulate TGF-β signalling and mitigate cavernosal fibrosis, as initially described by Ferrini et al. [16]. These findings were confirmed in animal studies conducted on rats [16]. A positive finding was echoed in a prospective series conducted by Lee et al. [17]. Glycemic optimisation augments host immunity and antibiotic efficacy. Our patient’s basal-bolus insulin regimen likely contributed to the rapid resolution. Accordingly, duplex ultrasound at 12 months and MRI at 24 months may be required to keep them in strict follow-up, particularly in people with diabetes.

In diabetic men with painful priapism, a tiny perineal abscess may herald a cavernosal abscess. Timely MRI and limited drainage, combined with culture-directed antibiotics and adjunctive PDE-5 inhibition, can eradicate the infection and preserve potency.

Limitations

The single-case design and six-month follow-up limit the external validity and assessment of late fibrosis. MRI may be unavailable in low-resource settings; however, patients with delayed presentation and large cavities may still require corporotomy, potentially introducing selection bias.

Take-home messages

Perineal abscess in a person with diabetes with priapism is virtually pathognomonic of cavernosal abscess. Rapid MRI delineation enables tissue-sparing drainage. Culture-directed antibiotics and meticulous irrigation are sufficient for small cavities without a tunical breach. PDE-5 inhibitors and glycaemic control are pragmatic, low-risk adjuncts that may safeguard erectile function.

## Conclusions

Spontaneous cavernosal abscess masquerading as priapism is rare yet curable. The cavernosal abscess should be suspected in diabetic men presenting with painful priapism and perineal tenderness. Early recognition through meticulous examination, prompt administration of broad-spectrum antibiotics, high-resolution MRI, and minimally invasive drainage can achieve rapid detumescence, eradicate infection, and preserve erectile function. Incorporation of perineal inspection and targeted imaging into priapism management algorithms may improve outcomes in this vulnerable population.
